# Incidence of Football and Futsal Injuries Among Youth in Malaysian Games 2018

**DOI:** 10.5704/MOJ.2003.005

**Published:** 2020-03

**Authors:** AH Ahmad-Shushami, S Abdul-Karim

**Affiliations:** Sports Medicine Unit, University of Malaya, Kuala Lumpur, Malaysia

**Keywords:** football, futsal, injury, Malaysian games, youth

## Abstract

**Introduction:**

Football and futsal were the main sports in the Malaysian Games. However, they were associated with a risk of injury. The purpose of the study was to analyse the incidence, circumstances, and characteristics of football and futsal injuries during the Malaysian Games of 2018.

**Materials and method:**

During the tournament, 14 teams participated in men’s football, 12 teams in men’s futsal and 11 teams in women’s futsal. The biannual event involved athletes aged under 21 years. A medical report form used by FIFA Medical Assessment and Research Centre (F-Marc), was provided to the physiotherapists and team doctors of all the teams to report all injuries after each match.

**Results:**

The response rate was 84.62% in football and 59.76% in futsal. A total of 48 injuries were reported from 26 football matches, equivalent to 64.64 injuries per 1000 match hours (95%CI 46.35 to 82.93). In futsal, a total of 48 injuries from 41 matches were reported, equivalent to 292.42 injuries per 1000 match hours (95% CI 209.7 to 375.14). The rate of injury in women futsal players was higher compared to men: 358.21 versus 247.04 injuries per 1000 match hours (p=0.224). Futsal recorded higher injuries per 1000 match hours than football (p<0.001).

**Conclusion:**

The rate of severe injury in futsal and football recorded in the study as compared to previous studies gave rise to serious concerns. Hence, there was an urgent need to pay more attention to injury prevention strategies.

## Introduction

Football is undoubtedly the most popular sports today and played widely around the globe, numbering approximately 400 million players in 208 countries^1^. The injury rate varied depending on the level of the tournament. It was also influenced by the characteristics of the players, such as age and gender. It was during the 1998 FIFA World Cup that the Fédération Internationale de Football Association (FIFA) started the initiative to document match injury^2^ systematically.

Based on the football injury surveillance involving twelve FIFA tournaments at the Olympics from 1998 to 2001, the total incidence recorded was 88.7 injuries per 1000 match hours or 2.7 injuries per match. The most frequently injured body parts were the ankle (17%) and thigh (16%)^2^. The overall incidence of injuries had reduced by 37% during the subsequent four editions of FIFA World Cup, from 2002 to 2014. During the FIFA World Cup 2014 in Brazil, a total of 104 injuries was recorded, which equalled to 58 injuries per 1000 match hours or 1.68 injuries per match. The reduction occurred possibly due to stricter refereeing and a better approach by players towards fair play^3^.

Futsal is one of the most popular indoor sports worldwide. It is a five-sided variant of football played on a different pitch surface and in a smaller sized field. A FIFA survey in 2006 showed that there were around one million registered futsal players worldwide^4^. Based on game analysis, futsal is a team sport characterised by multiple sprints and has more high-intensity phases than football^5, 6^.

Despite the popularity of football and futsal in Malaysia, systematic documentation regarding injury surveillance in both games, particularly among the youth, was limited. Although a study was conducted in 2010, describing the incidence of injury in futsal during the National Futsal League, it did not focus on young athletes. The study showed that the incidence of futsal injuries was 61 per 1000 player hours^7^. Incidences of injuries recorded during three editions of Futsal World Cups were higher, ranging from 168.5 to 236.0 injuries per 1000 player hours^8^. To date, there were no published data on football injury surveillance in Malaysia.

Given the paucity of injury-related research on young athletes, the study aimed to analyse the incidence and characteristics of football and futsal injuries among young athletes during the Malaysian Games of 2018. Injury surveillance study was an important first step in the implementation of injury prevention programs^9^. Such studies were crucial to ensure that the types and mechanism of injuries could be analysed, and prevention programs started at an early age. Hence, the study was essential in formulating injury prevention programmes in conjunction with protecting the health of athletes in Malaysia.

## Materials and Methods

The study was conducted during the 2018 Malaysian Games, the SUKMA Games, which was held from 12th to 22nd September 2018 in the state of Perak. There were 14 teams participating in men’s football, 12 teams in men’s futsal and 11 teams in women’s futsal. This biannual event involved athletes under 21 years of age While the football matches were held in four different stadiums, both the men’s and women’s futsal matches were conducted in one venue, the Manjung Indoor Sports Arena.

The data collection was conducted based on the method proposed by the Injury Consensus Group, which was established under the Federation Internationale de Football Association Medical Assessment and Research Centre (F-MARC)^10^. Before data collection, a briefing on how to fill up the injury report form was completed by the investigator to those involved in the study: the team doctor, the team physiotherapist and the tournament doctor. After each match, the team doctor or the physiotherapist of the participating teams was requested to document all injuries.

The injury report form as proposed by the F-MARC consensus, comprised of a single page on which all injuries sustained during a match were recorded. If there was no injury, the non-occurrence was also reported. According to the consensus statement^10^, an injury was defined as “any physical complaint sustained by a player that resulted from a football match or football training, irrespective of the need for medical attention or time loss from football activities”. An injury that resulted in a player receiving medical attention was referred to as a “medical attention” injury and an injury that resulted in a player being unable to take a full part in future football training or match play as a “time loss” injury. Details of the injury documented were the time of injury, the body part involved and the type, mechanism and sequelae of the injury. The completed forms were collected after each match by the investigator. All efforts were taken to ensure the confidentiality of the information collected by which all the data were anonymous and kept by the investigator immediately after each match.

Response rate, exposure time and incidences were computed as proposed by the F-MARC consensus and previous studies^2, 3, 7, 10^. The injury rates were stated as the number of injuries per match, and the number of injuries per 1000 match hours. The exposure factor was the duration of time during which the participant was exposed to the injury risk. In the study, the exposure time was only recorded from match exposure. For football, the total match exposure was calculated as 11 players multiplied by 1.5 hours multiplied by the number of returned injury report forms^10^. In the case of futsal, the total match exposure was calculated as five players multiplied by 40 or 60 minutes multiplied by the number of returned injury report forms^10^. Extra time allocated for a match and the reduction in the number of players on the field were not considered. Therefore, injuries per 1000 match hours were computed as the number of injuries multiplied by 1000 divided by total match hours. For incidence rates, 95% CI was calculated as the incidence ±1.96 times the incidence divided by the square root of the number of injuries^10^. The severity of injuries was classified as slight (1-3 days’ absence), minor (4-7 days), moderate (8-28 days), and major (more than 28 days)^10^.

Microsoft Excel and SPSS were used to process the data. Data were analysed descriptively with normality test conducted using the Shapiro-Wilk test. The statistical analysis applied were frequencies, and Mann-Whitney U test as the injury incidences between the teams were not normally distributed. The significance value was accepted at the 5% level. The study objective and procedures were explained clearly to all the participants with the patient’s information sheet. They were required to sign the informed consent form before participating in the study. For participants who were less than 18-years old, written consent was obtained from parents or guardians. The ethical approval to proceed with the study was granted by the Medical Research and Ethics Committee (MREC) of the University of Malaya Medical Centre.

## Results

A total of 252 players from 14 teams participated in football during the Malaysian Games 2018. The median age group for injured players was 20±2. In total, 44 out of 52 daily medical report forms were returned with a response rate of 84.62%. Therefore, the total match exposure calculated was 726 hours (Table I). The training exposure was not included in the study.

**Table I:** Number of matches, response rate, severity and incidence of injury in football and futsal.

Estimated duration of absence from sports	Football	Men’s futsal	Women’s Futsal
Matches(n)	26	20	21
Returned Match Reports (n)	44/52	29/40	20/42
Response Rate (%	84.62%	72.50%	47.62%
Match Hours Documented	726	97.15	67
All injuries(n)	48	24	24
Injuries per 1000 match hours (95% CI)	66.12	247.04	358.21
	(47.42, 84.84)	(148.21, 345.88)	(214.9 to 501.52)
Injuries per match (95% CI)	2.18 (1.56, 2.8)	1.66 (1.33 to 1.96)	2.4 (1.83 to 2.97)

Where football was concerned, 48 injuries were recorded during the competition. The total injury incidence was 2.18 injuries per match with 95% CI [1.56,2.8] or 66.12 injuries per 1000 match hours, with 95% CI [47.42,84.84] (Table I). Approximately, a third of the injured players (n=15 or 31.25%) were able to return to play within the same day. Time loss injuries were recorded as 45.45 injuries per 1000 hours, 95% CI [42.89, 58.31] or 1.5 injuries per match, 95% CI [1.08,1.92]. A total of 25 (52.08%) slight injuries were recorded which led to a time loss from sport, estimated as 13 days. There were four (8.33%) minor injuries which caused an absence of 4-7 days and only one (2.08%) moderate injury resulting in time loss of 14 days. In the case of three injuries, a shoulder dislocation, an ankle ligament rupture and a hamstring tear, the estimated duration of absence was more than four weeks (Table II).

**Table II:** Estimated duration of absence from sports

Estimated duration of absence from sports	Football	Men’s futsal	Women’s Futsal
0 days	15 (31.25%)	2 (8.33%)	3 (12.5%)
1-3 days	25 (52.08%)	7 (29.17%)	10 (41.67)
4-7 days	4 (8.33%)	1 (4.16%)	0
8-28 days	1 (2.08%)	3 (12.5%)	6 (25%)
>29 days	3 (6.25%)	3 (12.5%)	5 (20.83%)
Not specified	0	0	0
Injuries with the subsequent absence	33	14	21

As can be observed from Table III, the injuries most frequently involved was the lower extremities (n=37; 77.08%) followed by the head/neck (n=6; 12.55%), trunk (n=3; 6.25%) and upper extremities (n=1; 2.08%). The commonest injury diagnosed was strain (n= 21; 43.75%) followed by contusion (n=12;25%) and ligament sprain (n=6,12.5%). 14.58% of the injuries in football were recurrent injuries.

**Table III:** Location, type and mechanism of injury in football and futsal

Location	Football	Men’s Futsal	Women’s futsal
Head, face, neck	7(14.58%)	1(4%)	0
Chest	0	1(4%)	1(3.7%)
Upper extremity including shoulder	1(2.08%)	1(4%)	5(18.52%)
Abdomen	0	0	2(4.07%)
Back	3(6.25%)	0	1(3.7%)
Pelvic	1(2.08%)	0	0
Hip/Groin	5(10.41%)	4(16%)	0
Thigh	8(16.67%)	6(24%)	2(4.07%)
Knee	5(10.42%)	5(20%)	10(37.04%)
Lower leg	8(16.67%)	2(8%)	1(3.7%)
Ankle	9(18.75)	4(16%)	4(14.81%)
Foot/toe	1(0.02%)	1(4&	1(3.7%)
Total	48	25*	27*
**Type of injury**			
Concussion	3(6.25%)	0	0
Fracture	0	0	1(3.85%)
Rupture of tendon or ligament/meniscus lesion	1(2.08%)	1(4%)	2(7.69%)
Sprain	7(14.58%)	9(36%)	11(42.31%)
Strain/muscle fibre rupture	21(43.75%)	11(44%)	5(19.23%)
Contusion	12(25%)	3(12%)	5(19.235)
Laceration/abrasion/blister	3(6.25%)	1(4%)	2(7.69%)
Dislocation	1(2.08%)	0	0
Total	48	25	26
Missing	0	0	1
**Mechanism**			
Non-contact injuries	16(33.33%)	9(37.5%)	5(20.83%)
Contact injuries	32(66.67%)	12(50%)	19(79.17%)
Unspecified	0	3	0
Contact injuries caused by foul	20	4	6
Foul sanctioned by the referee	16	4	6
**Recurrent injury**	7(14.58%)	6(24%)	7(25.92%)

*One injury affected two body parts.

Two-thirds of the injuries in football (n=32; 66.67%) were caused by contact, and more than half (20/32) was a result of foul play based on referee decision. Ankle sprain was the most frequent diagnosis (n=7), and all ankle sprains were bound to lead to an absence of play. Most (85.71%) ankle sprains were incurred due to contact. Similarly, almost all head and neck injuries were caused by contact (83.3%); however, only two were classified as foul play. Head and neck injuries were diagnosed as concussion (n=3), laceration (n=1), contusion (n=1) and neck sprain (n=1).

For futsal, 352 players (192 men and 160 women) from 22 teams (12 male and ten female) took part in the Malaysian Games 2018. All the players who participated in this edition were amateurs. The response rate was much lower than football, whereby 59.76 % of the daily medical report forms were returned while the remaining medical reports were missing. The response was lower among the women’s teams, with only 47.62% of total expected injury reports being returned. On the other hand, the men’s team response rate was much better with a turning in of 29 out of 40 medical reports or 72.5%. Hence, the total match hours for both men’s and women’s teams were 164.15.

In total, 48 injuries were reported throughout the futsal competition. Total player hours documented were 164.15. The overall incidence of injuries was 1.96 injuries per match, and 292.42 injuries per 1000 match hours. The rate of injury amongst women was higher at 2.4 injuries per match or 358.21 injuries per 1000 match hours compared to men who had 1.66 injuries per match or 247.04.3 injuries per 1000 match hours (Table I). However, the difference was not statistically significant (p-value = 0.224).

The injuries most frequently affected were the lower extremities (n=36; 69.23%). Where the lower limbs were concerned, the knee was the most frequently injured body part (n=14,26.92%), followed by the thigh (n=8,15.38%) and ankle (n=7,13.46%). The commonest injury diagnosed was sprain (n=20; 38.46.36%) followed by muscle strains (n=16,30.77%) and contusion (n=8,16.67%). In futsal, almost two-thirds (n=31; 64.58.36%) of injuries resulted from contact with other players while the remaining (n=14,29.17%) were non-contact injury. Contrary to football (41.6%), in futsal, a smaller percentage (32.2%) of contact injuries or 20.83% (10/48) of all injuries resulted from foul play as decided by the referee (Table III). There was a higher rate of recurrent injuries in futsal (24% in men and 25.92% in women) compared to men’s football (14.58%) (Table III).

Data about time loss from the sport was recorded in 40 (83.33%) injuries while the other eight (16.67%) injuries were not specified. Only five injuries (10.42%) did not result in time loss. Approximately one-third of the injuries (n=17; 35.41%) resulted in absence from the sport for 1-3 days, one injury (2.08%) caused the absence of 4-7 days, and nine (18.75%) resulted in the absence of 8-28 days. Eight injuries were predicted to have time loss of more than four weeks: three knee ligamentous injury, one meniscus injury, three ankles ligamentous injury and an adductor muscle tear (Table III).

## Discussion

This was the first prospective study conducted in Malaysia on injury surveillance among young football players during a local tournament, the Malaysian Games. The main objective was to investigate football and futsal related injuries sustained by players during the Games. It was the second study related to futsal in Malaysia as the first one was conducted in 20107.

In this study, the response rate for football was 84.62% and 59.76% for futsal. According to the injury surveillance of various FIFA tournaments from 1998 to 2001, the average response rate had been 84%, ranging from 47% to 100%. This showed that the method of the data collection had high compliance as most of those studies demonstrated response rates up to 100%^3^. It had been suggested that response rates of less than 70% should be cautiously interpreted^2^. From the response rate of this study, we could conclude that the rate for football was slightly above average while that for futsal reflected a below average response. The response rate of 47.5% as recorded for the women futsal teams was comparable to that of the FIFA Confederations Cup in 19992. The response could reflect the standard of care among football and futsal players in Malaysia. Hence, there was a need to study further the knowledge and attitude of medical staff who provided care for athletes in Malaysia.

The overall incidence of football injuries in the Malaysian Games was lower when compared with that reported for the previous edition of Under 20 World Cups^2^. The difference in the level of playing level could be one factor contributing to the lower injury rate. It had been suggested by van Beijsterveldt *et al* (2015) that higher match intensity at a more advanced level of tournaments could explain the increased injury rate among professional players^11^. However, closer scrutiny showed that the rate of severe injury was higher during the Malaysian Games compared to previous Under 20 World Cups (6.25% vs 2%)^12^.

The overall injuries for futsal reported for the present study were higher compared to the previous surveys^7, 8, 13^. The total injury incidence was 292.42 per 1000 match hours compared to 168.5 - 236 injuries per 1000 match hours during three editions of futsal world cup from 2000 to 20088. More injuries were recorded for women’s futsal games compared to men: 358.21 versus 247.04 injuries per 1000 match hours, respectively. However, the former was not statistically significant (p-value=0.224). These findings were similar to those found from a survey done on the Iran futsal national team, which recorded higher injury in women compared to men^13^. This could be attributed to a lower level of physical fitness in female players which included neuromuscular control, balance, strength and agility. A lower level of physical fitness was one of the main risk factors which could contribute to sports injuries^14, 15^. In previous surveys during FIFA tournaments from 1998 to 2012, the incidence of injuries between male and female players was similar when comparisons were made at the same level of competition. Other tournaments had reported lower injuries among female players^12^.

The study showed that in comparison with football, futsal tournaments had a higher number of injuries : 66.12 versus 292.42 injuries per 1000 match hours. There was also a significant difference between the rate of injuries in both sports (p<0.001). This finding was supported by a study during the FIFA and Olympic Tournaments^2^. Several factors could have contributed to the difference: the characteristics of futsal, a game played at higher speed and intensity; the smaller field or area of play, resulting in more collisions; and the different playing surface compared to football8.

The incidence of moderate to severe injuries in futsal was higher than that in football. In futsal, 35.4% (n=17) of injuries were reported as moderate to severe compared to 8.3% (n=4) injuries in football. The high number of moderate to severe football and futsal injuries in this tournament was a cause for concern when compared to previous injury surveillance^7, 8, 13^. Therefore, a more collaborative effort would be needed from various stakeholders to enhance players’ welfare by focusing on improving medical care and implementing more effective injury prevention programmes.

In the study, three (6.25%) football players were reported to have suffered a concussion. In comparison, previous injury surveillance reported a total of 12 concussions during five editions of the FIFA World Cup, from 1998 to 20143. It was crucial to educate the medical team involved in the right approach to handle concussion, particularly regarding the recognition and removal of the player from the game. Another important aspect was understanding the recommended protocol on a return to play decision for concussion.

It was important to have a complete understanding of the multifactorial nature of sports injuries. Although the injury might be caused by a single inciting event, it could have resulted from a complex interaction between various internal risk factors of biological maturity, maximal aerobic capacity, muscle strength and previous injuries and the external risk factors of coaching style, refereeing quality, weather and pitch condition^16^. History of the previous injury was the most consistent risk factor for a newer injury to the body parts^17, 18^. In the study, there were 14.58% recurrent injuries in football and a higher rate of recurrence in futsal with 24% for men’s futsal and 25.92% for women’s futsal. A further study was needed to explore the risk factors of injuries among Malaysian youth footballers.

The study was not without some limitations. First, only injuries during the match were recorded. Injuries incurred during training were not recorded. Players who had illness or diseases were also not registered. The injury documentation depended entirely on the report by the team physiotherapist or the team doctor. Therefore, the possibilities of underreporting and making mistakes while recording the details of injuries could not be excluded as some teams did not have a team physician. However, the physiotherapists were the first-line medical personnel in their respective teams, and thus, they were the best people to document the injuries. The presence of doctors who were expert in sports injury in the tournament medical team could also improve the reliability of diagnosis and treatment for the players. The other issue was the involvement of different physiotherapists in data collection, which could affect the consistency of the information. It was believed that the problem had been minimised by using a recommended form with exact definitions and fixed response options. Furthermore, briefing on data collection was done to every physiotherapist and team physician involved.

## Conclusion

This study which focused on injuries incurred by young players during tournaments had shown that during the Malaysian Games, the injury rate of futsal was higher compared to that of football. Women futsal players recorded a higher injury rate compared to their male counterparts though statistically, it was not significant. Interestingly, a high percentage of severe injury among players for both futsal and football matches were recorded for the tournament. Given that previous studies^7, 8, 13^ showed injury surveillance rates much lower than that reported for this study, more attention should be given to injury prevention strategies and to improving medical care for players, especially the youth.
